# Co-ordinated control of the Aurora B abscission checkpoint by PKCε complex assembly, midbody recruitment and retention

**DOI:** 10.1042/BCJ20210283

**Published:** 2021-06-18

**Authors:** Lisa Watson, Tanya N. Soliman, Khalil Davis, Joanna Kelly, Nicola Lockwood, Xiaoping Yang, Steven Lynham, John D. Scott, Victoria Crossland, Neil Q. McDonald, David J. Mann, Alan Armstrong, Ulrike Eggert, Peter J. Parker

**Affiliations:** 1 Protein Phosphorylation Laboratory, Francis Crick Institute, Midland Road, London NE1 1AT, U.K.; 2 Proteomics Facility, King's College London, Denmark Hill Campus, London SE5 9NU, U.K.; 3 Department of Pharmacology, University of Washington, Seattle, WA 98195, U.S.A.; 4 Signalling and Structural Biology Laboratory, Francis Crick Institute, Midland Road, London NE1 1AT, U.K.; 5 Institute of Structural and Molecular Biology, Department of Biological Sciences, Birkbeck College, London WC1E 7HX, U.K.; 6 Department of Life Sciences, Imperial College London, South Kensington Campus, London SW7 2AZ, U.K.; 7 Department of Chemistry, Imperial College London White City Campus, London W12 0BZ, U.K.; 8 Randall Centre for Cell and Molecular Biophysics, School of Basic and Medical Biosciences, King's College London, London SE1 1UL, U.K.; 9 Department of Chemistry, King's College London, London SE1 1UL, U.K.; 10 CRUK KHP Centre, School of Cancer and Pharmaceutical Sciences, King's College London, London SE1 1UL, U.K.

**Keywords:** 14-3-3, abscission checkpoint, aurora kinases, midbody, protein kinase C

## Abstract

A requirement for PKCε in exiting from the Aurora B dependent abscission checkpoint is associated with events at the midbody, however, the recruitment, retention and action of PKCε in this compartment are poorly understood. Here, the prerequisite for 14-3-3 complex assembly in this pathway is directly linked to the phosphorylation of Aurora B S227 at the midbody. However, while essential for PKCε control of Aurora B, 14-3-3 association is shown to be unnecessary for the activity-dependent enrichment of PKCε at the midbody. This localisation is demonstrated to be an autonomous property of the inactive PKCε D532N mutant, consistent with activity-dependent dissociation. The C1A and C1B domains are necessary for this localisation, while the C2 domain and inter-C1 domain (IC1D) are necessary for retention at the midbody. Furthermore, it is shown that while the IC1D mutant retains 14-3-3 complex proficiency, it does not support Aurora B phosphorylation, nor rescues division failure observed with knockdown of endogenous PKCε. It is concluded that the concerted action of multiple independent events facilitates PKCε phosphorylation of Aurora B at the midbody to control exit from the abscission checkpoint.

## Introduction

Checkpoints acting through the cell cycle serve as critical safeguards of genome integrity and determine the timing of progression. Amongst these is the Aurora B (S/T protein kinase) dependent abscission checkpoint that acts at the ultimate stage of cell division to control the timing of the final act of division and the separation of presumptive daughter cells (recently reviewed [1,2]). This checkpoint was originally identified in budding yeast and termed the NoCut Pathway [3], requiring Ipl1 (Aurora) to delay cytokinesis, so protecting from chromosome breakage and likely associated with chromatin in the midzone. A related pathway has been shown to operate in mammals, under the control of Aurora B, where chromatin trapped in the intercellular canal activates the checkpoint and failure leads to chromatin breakage [3–6]. The maintenance of Aurora B activity is required to both engage the checkpoint and sustain intercellular canals, with furrow regression and tetraploidization ensuing on Aurora B inhibition [6]. Functionally the relationship to the abscission machinery is afforded by the Aurora B phosphorylation of CHMP4C at the midbody [5] and the ANCHR-dependent sequestration of Vps4 [7].

Aurora B is a component of the chromosome passenger complex (CPC) alongside INCENP, Borealin and Survivin, and plays an essential role in chromosome segregation at multiple checkpoints (see [8,9]). After quality controlling microtubule engagement of kinetochores (recently reviewed [10]), the CPC localises to the central spindle, relocates to the midzone and subsequently to the equatorial cortex [11,12]. This relocation to the cortex is under cdk1 control [13,14] and is reliant on MKLP2 and INCENP interactions with microtubules as well as INCENP-actin interaction at the cortex [15–17]. On furrow ingression the CPC then accumulates at the midbody in a Survivin dependent manner [18] and under the control of ATM/chk2 [19]; here Aurora B determines the timing of abscission [6].

PKCε is a member of the protein kinase C family of S/T protein kinases, retaining a conserved C2 domain (non-Ca^2+^ binding), an archetypal pair of diacylglycerol binding C1A and C1B domains, linked to its C-terminal catalytic domain (reviewed [20]). It has been implicated in a series of cell cycle controls associated with the dysfunction of a G2 arrest, operating both prior to anaphase onset and at cytokinesis [21–24]. Specifically, it has been shown that exit from the abscission checkpoint requires Aurora B phosphorylation by PKCε on S227 in the activation loop of the kinase, altering Aurora B specificity and promoting the phosphorylation of Borealin on S165 [24]. This phosphorylation occurs at the midbody and recent evidence suggests this switch in Aurora B specificity operates to determine the timing of checkpoint exit in concert with the midbody recruitment of PP1 via RIF1 and dephosphorylation of CHMP4C [25]. In contrast with the detailed insights into Aurora B recruitment to this compartment through its central role as the catalytic module of the CPC (reviewed [9,26]), the localisation of PKCε at the midbody is poorly understood, beyond the observation that it is impacted by its own activity, with stable accumulation occurring only under conditions of its inhibition [27]. In transformed cells that engage the Aurora B checkpoint, division failure has been associated with knockdown of PKCε and also with the failure of PKCε to complex 14-3-3 [27].

The scaffold protein 14-3-3 family, comprises seven proteins that as obligate dimers assemble with partners generally through recognition of sequence-specific phosphorylation sites, activating, inhibiting and sequestering partners in compartments (see [28]). Assembly of the PKCε/14-3-3 complex is an activating event locking a lipid-independent conformer of PKCε [27]. We previously reported that knockdown of endogenous PKCε or expression of mutants which could not form a PKCε/14-3-3 complex induced a telophase delay, consequently failing cytokinesis resulting in an accumulation of binucleated cells [27]. A similar phenotype was also observed after the expression of a dominant-negative mutant of 14-3-3 [27]. Whether this activated PKCε/14-3-3 complex is required for Aurora B phosphorylation and if so, how this relates to recruitment and activity-sensitive retention at the midbody is unknown. Equally, the combined requirements for all these steps in relation to the successful completion of cell division is not understood.

Here, we address PKCε relationships and demonstrate that 14-3-3 complex formation is required for Aurora B S227 phosphorylation, but not for recruitment and retention of PKCε at the midbody, which appear to require C1A + C1B domains and C2/inter-C1 domains. Mutations compromising any of these individual PKCε attributes phenocopy PKCε loss and division failure, indicating that these multiple events collectively impact Aurora B phosphorylation and the exit from the cytokinesis checkpoint.

## Materials and methods

### Reagents

All reagents were purchased from Sigma unless otherwise specified. Cross-linking amino acid AbK (DiAzK) was purchased from siChem (SC-8034) and the inhibitor Na-PP1 was generated in house. PMA was purchased from Alexis, and sn-1,2-didecanoylglycerol (diC10) was purchased from Avanti Polar Lipids.

### Cell lines, DNA plasmid transfection and siRNA transfection

All cell lines were cultured at 37°C and 10% CO_2_ in Dulbecco's modified Eagle's medium (DMEM) supplemented with 10% FBS and 1% Pen/Strep (Gibco). Cell lines were obtained from the American Type Culture Collection unless otherwise stated and all cell lines were authenticated through the Francis Crick Institute Cell Services. Tetracycline-inducible DLD1 (a gift from Prof. Stephen Taylor) were generated using the T-Rex Flp-In system (Invitrogen) according to the manufacturer's instructions. To induce expression of GFP-PKCε, cells were cultured in DMEM containing 10% FBS and tetracycline (100 ng ml^−1^) for 16–24 h before assay. Cells were treated for 1 h with inhibitors unless otherwise stated. Cells were transfected with siRNA for 72 h using Lipofectamine RNAiMAX reagent (Invitrogen) for HEK293T cells or Lullaby siRNA Transfection Reagent (OZ Biosciences) for DLD1 cells, according to the manufacturer's instructions. The following siRNAs were purchased from Dharmacon and used at 20 nM: Scrambled control pool (D-001206-13-20 5′-UAGCGAUAAACACAUCAA-3′;5′-UAAGGCUAUGAAGAGAUAC-3′;5′-AUGUAUUGGCCUGUAUUAG-3′;5′-AUGAACGUGAAUUGCUCAA-3′); PKCε si1 (D-004653-01 5′-GGGCAAAGAUGAAGUAUAU-3′). For HEK293T cells, after the required change of media after 6 h of RNAiMAX incubation, cells were transiently transfected with purified plasmid DNA using the Lipofectamine LTX reagent (Thermo Fisher Scientific) in a 1 : 3 DNA : Lipofectamine LTX ratio, and then incubated for a further 66 h before analysis. For DLD1 cells, 24 h post-transfection with siRNA, doxycycline was added to the media at a concentration of 1 µg/ml and cells were incubated for 48 h before analysis to allow expression of the plasmid to take place.

### Cell cycle synchronisation

Cells to be synchronised were arrested with 2 mM thymidine for 16 h, and cells were then washed 3× with PBS and released with complete DMEM for 8 h. To arrest cells in mitosis, cells were blocked with 500 nM nocodazole for 14–16 h and harvested by mitotic shake-off. The harvested cells were transferred to a 15 ml Falcon tube and centrifuged for 5 min at 1000 rpm. Cells were resuspended in complete DMEM and then replated for analysis.

### Genetic incorporation of AbK and photocrosslinking

The site on the protein of interest where AbK is inserted was first mutated to TAG in the expression plasmid followed by co-transfection in a 1 : 1 ratio with a plasmid generously provided by the Schultz group [29] that encodes both an aaRS optimised to recognise AbK, and the tRNA^Pyl^ which recognises the UAG codon. Cell media was replaced with DMEM containing 1 mM AbK prior to the addition of transfection complexes. AbK was added to DMEM from a 100 mM stock in dH_2_O, before being passed through a 0.22 μm filter unit for sterilisation. Transfected cells were incubated and irradiated for 10 min on ice with 365 nm UV light using a Stratalinker 2400 (Stratagene). Cells were then washed briefly with ice-cold PBS and processed for western blot analysis.

### Co-immunoprecipitation and immunoblotting

Lysates were obtained by washing cells 2× with ice-cold PBS before the addition of RIPA buffer (1% Triton X-100, 1% Sodium deoxycholate, 0.1% SDS, 150 mM NaCl, 1 mM EDTA, 50 mM Tris (pH 7.4)) supplemented with PhosStop and Complete EDTA-free Protease Inhibitor Cocktail (Roche). Following 10 min of incubation on ice, lysates were sonicated and cleared through centrifugation at 13 200 rpm at 4°C for 10 min. 2× LDS sample buffer (Invitrogen) was subsequently added to the lysates with a final concentration of 100 mM DTT, followed by boiling at 95°C for 5 min. In the case of GFP immunoprecipitations, Protein A/G PLUS-agarose beads (Santa Cruz, product code sc-2003) were first washed and used to pre-clear the lysates to reduce unspecific binding to beads, and anti-GFP DARPin beads (in house) were used to isolate GFP-tagged proteins. The resulting samples were then loaded to NuSep Tris-HEPES (Thermo) polyacrylamide gels, separated by SDS–PAGE and transferred to PVDF membranes. Membranes were blocked for 1 h at room temperature in buffer containing 3% BSA for the following antibodies: Rabbit PKCε (Santa Cruz, sc-214), Rabbit PKCε pS346 (In house), mouse pan-14-3-3 (Santa Cruz, sc-1657) mouse GAPDH (Millipore, MAB374). Antibodies were subsequently detected using HRP-conjugated secondary antibody (GE Healthcare) and Luminata Classico ECL reagent (Millipore) and imaged using an ImageQuant LAS 4000 (GE) set to detect chemiluminescence.

### Mass spectrometry analysis

Samples were separated by SDS–PAGE and stained with Coomassie blue. Upon identification of the bands to be analysed, these were excised and subjected to destaining. In-gel reduction, alkylation and digestion with AspN was performed on the excised gel bands. Digestion was carried out overnight at room temperature following an initial incubation at 37°C for 2 h. Cysteine residues were reduced with dithiothreitol and derivatised by treatment with iodoacetamide to form stable carbamidomethyl derivatives. Chromatographic separation was performed using an Ultimate 3000 NanoLC system (ThermoFisher Scientific). Peptides were resolved by reverse phase chromatography on a 75 µm × 15 cm C18 column using a linear gradient of water in 0.1% formic acid (A) and 80% acetonitrile in 0.1% formic acid (B). The gradient was delivered to elute the peptides at a flow rate of 250 nl/min over 90 min.

Eluates were ionised by electrospray ionisation using an Orbitrap-Fusion-Lumos Tribrid Mass Spectrometer (ThermoFisher Scientific) operating under Xcalibur v4.1. For phosphoproteomics, the instrument was programmed to acquire using an advanced peak determination (APD) algorithm in a higher-energy collisional dissociation (HCD) mode and a targeted parallel reaction monitoring (PRM) method. For identification of cross-linked proteins, the instrument was programmed to acquire using a ‘Universal’ method in a collision-induced dissociation (CID) mode. Raw mass spectrometry data were processed into peak list files using Proteome Discover (PD v2.2, ThermoFisher Scientific), and processed data were searched using Sequest search engine embedded in PD v2.2 against Homo sapiens databases downloaded from Uniprot and PKCε-AbK sequences. For phosphorylation site identification, the spectra obtained were subjected to manual analysis to eliminate false-positive results.

### Fluorescence polarisation

FAM-tagged PKCε phosphopeptides were resuspended in FP buffer (50 mM Tris (pH 7.5), 150 mM NaCl, 0.5 mM TCEP), and concentrations were determined and corrected by measuring absorbance using a V-550 UV–Vis Spectrophotometer (Jasco). The Beer–Lambert law equation was used to calculate the exact concentration.

A series of twenty two-fold dilutions of 14-3-3, beginning at 175 µM, was made by sequentially diluting 1 volume 14-3-3 with 1 volume FP buffer. Ten microlitre of each 14-3-3 concentration was added to a well of a 384-well microplate (black, low volume, flat bottom, Corning). PKCε phosphopeptide was then added to each well to have a final concentration of 10 nM peptide, in a total volume of 20 µl per reaction. The microplate was placed in a CLARIOstar plate reader (BMG Labtech), and the contents of each plate were mixed for 15 s and anisotropy measurements taken at 25°C. A well with 10 nM pSer346 peptide was used to adjust the gain settings for parallel and perpendicular fluorescence measurements, based on the anisotropy *r* = 0.03, obtained from measurements on a conventional fluorescence spectrometer (Jasco FP8500, equipped with automated polarisers). Data were then plotted and analysed using Graphpad Prism software. The following equation was used for the calculation of $K_{d}:r=r_{\;f\;\mathrm{ree}}+(r_{\mathrm{complex}}-r_{\mathrm{free}})\times (K_{d}+X+F-\sqrt{{\left(K_{d}+X+F\right)}^{2}}-4\times F\times X)/$ (2×F) (*r*: measured anisotropy, *r*_free_: anisotropy of the unbound (or free) peptide, *r*_complex_: anisotropy of the peptide:14-3-3 complex, *K*_d_: dissociation constant, F: constant concentration of FAM-labelled peptide, X: concentration of 14-3-3). For pSer346/pSer368 and pSer346/pSer350/pSer368 peptides that bind simultaneously to both sites in the 14-3-3 dimer in a 1 : 1 peptide-to-dimer stoichiometry [30], the concentration of dimers was used. For all other peptides, where two peptides bind to one 14-3-3 dimer (2 : 1 stoichiometry), the concentration of 14-3-3 monomers was used.

### Immunofluorescence microscopy

Cells were grown on 13 mm glass coverslips and simultaneously fixed and permeabilized with PHEM-TX buffer (60 mM PIPES (pH 6.8), 25 mM HEPES (pH7.4), 10 mM EGTA (pH 8), 4 mM MgSO_4_, 4% paraformaldehyde and 0.1% Triton X-100) for 20 min. Cells were then incubated in blocking buffer (3% BSA in PBS) and probed using the following primary antibodies: rabbit Aurora B pS227 (in house, 1 : 100), mouse α-tubulin (in house, 1 : 1000). Primary antibodies were detected using Alexa Fluor-conjugated secondary antibodies (Life Technologies) and were all used diluted in blocking buffer: donkey anti-rabbit Alexa Fluor 647 and goat anti-mouse Alexa Fluor 555. All coverslips were mounted using ProLong Gold Diamond with DAPI (Invitrogen). All the static images were acquired using an inverted laser scanning confocal microscope (Carl Zeiss LSM 780) equipped with a 40× Plan-APOCHROMAT DIC oil-immersion objective. Image analysis was carried out using the ZEN analysis software. The variation in expression precluded midbody/cytoplasmic ratio determination as a valid approach to scoring localisation. To ensure consistency, initial scoring of midbody localisation for C1A/C1B mutants was carried out visually on mutant expression and treatment blinded samples; subsequent analyses were unblinded. Scoring recruitment to midbody/tubulin connected structures is shown as a percentage of the total number of such structures observed across multiple fields.

### Fluorescence recovery after photobleaching

Live-cell fluorescence recovery after photobleaching (FRAP) experiments were performed using an inverted laser scanning confocal microscope (LSM 510, Carl Zeiss MicroImaging GmbH, Jena, Germany), with fully open pinhole, using a Plan-Apochromat oil-immersion objective (×63, NA 1.4). The microscope was encased inside an environmental polymethylmethacrylate case maintained at a constant temperature of 37°C, while the dishes containing the cells were maintained under a constant 5% CO_2_ flow. GFP-PKCε M486A cells were incubated with NaPP1 (4 μM) for 10 min. Each FRAP experiment comprised a sequence of 300 frames (128 × 128, pixel dwell time of 6.40 μs, pixel size 0.37 μm) including 10 pre-bleached frames. The bleaching process consisted of 100 iterations at 100% of bleach pulse (488 nm laser excitation, 30 mW) on a circular region of two pixels in area, within the midbody or membrane. Recovery was monitored by capturing a further 290 scans after the photobleach event. Quantification was performed using the FRAP module included in the LSM software (Carl Zeiss) plotting the graphs to a best fit double exponential model *I = I*_0_*_ _− I*_1_*e^−t/T^*^1* *^*− I*_2_*e^−t/T^*^2^ where *I*_0_ is the intensity before bleach, *I*_1_ and *I*_2_ are the mobile fractions and *T*_1_ and *T*_2_ the time constants.

### Statistical analysis

Statistical analyses of results were calculated using Prism 7 software (version 7.0c) (GraphPad). For experiments where comparisons were made between more than two conditions, a one-way analysis of variance (ANOVA) was used. All other cases utilised an unpaired *t*-test for analysis. The level of statistical significance in the results is represented as follows: **** = *P *≤ 0.0001; *** = *P *≤ 0.001; ** = *P *≤ 0.01; * = *P *≤ 0.05; ns (not significant) = *P *> 0.05.

## Results

### PKCε-14-3-3 complex formation is required for Aurora B S227 phosphorylation at the midbody

**Figure 1.**
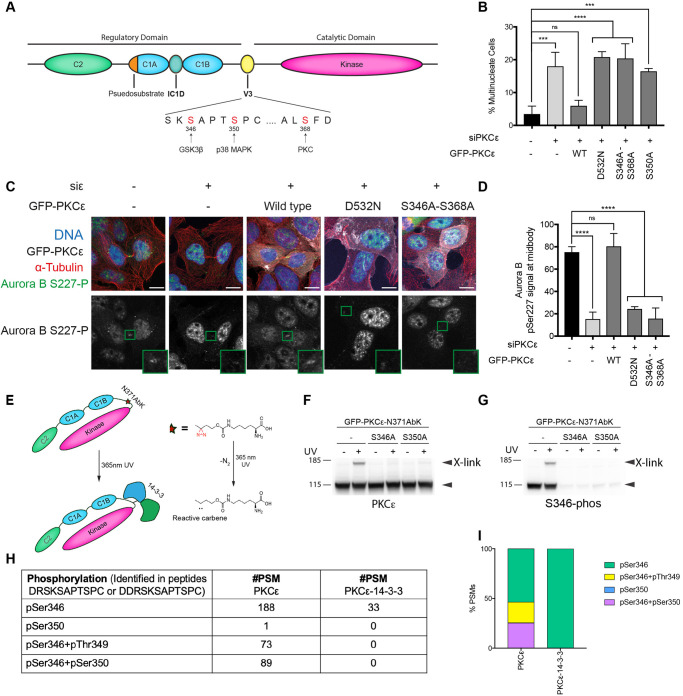
A S350 dephosphorylated PKCε-14-3-3 complex is required for Aurora B S227 phosphorylation at the midbody. (**A**) Schematic representing the organisation of PKCε, highlighting the three serine residues of the V3 domain required for 14-3-3 binding denoted in red alongside the kinases responsible for their phosphorylation. (**B**) Quantitation of multinucleate cells upon treatment of cells with non-targeting siRNA (control) or PKCε siRNA, with expression of siRNA-resistant mouse GFP-PKCε, GFP-PKCε-D532N, GFP-PKCε-S346A-S368A or GFP-PKCε-S350A. Values plotted are means for *n *= 3 experiments, with error bars denoting SD. Two-hundred cells were counted per condition per experiment. One-way analysis of variance: **** = *P *≤ 0.0001; *** = *P *≤ 0.001; ns (not significant) = *P *> 0.05. (**C**) Representative immunofluorescence images of DLD1 FRT-TREx cells treated with non-targeting siRNA (control) or PKCε siRNA, with expression of either siRNA-resistant mouse GFP-PKCε, GFP-PKCε-D532N or GFP-PKCε-S346A-S368A induced with doxycycline. Cells stained for DNA (blue), tubulin (red) and Aurora B Ser227 phosphorylation (green), with GFP-PKCε in white if expressed. The Aurora B S227 phosphorylation is highlighted in the lower greyscale panels with the midbody region indicated expanded for each image. Scale bar = 20µm. (**D**) Quantitation of cells with Aurora B pSer227 signal at the midbody. DLD1 FRT-TREx cells with inducible expression of GFP-PKCε, GFP-PKCε-D532N or GFP-PKCε-S346A-S368A were treated with either non-targeting siRNA (control), PKCε siRNA or PKCε siRNA and doxycycline to induce expression of siRNA-resistant mouse GFP-PKCε constructs, and the Aurora B pSer227 signal at the midbody quantified. Values plotted are means for *n *= 3 experiments, with error bars denoting SD. Twenty cells with midbodies were counted per condition per experiment. One-way analysis of variance: **** = *P *≤ 0.0001; ns (not significant) = *P *> 0.05. (**E**) Schematic of incorporation of the diazirine amino acid AbK (red star) into the V3 domain (N371AbK) of PKCε. (**F**–**G**) Western blots probed with (**F**) PKCε antibody and (**G**) phosphoSer346 antibody, showing cross-linking in HEK293T cells expressing GFP-PKCε-N371AbK, GFP-PKCε-N371AbK-S346A or GFP-PKCε-N371AbK-S350A upon exposure to 365 nm UV for 10 min. The upper and lower arrowheads indicate cross-linked (X-link) PKCε and uncross-linked PKCε, respectively. Western blot images are a representative example of three independent experiments. (**H**) Table showing the number of PKCε peptide spectrum matches (#PSM) identified in the mass spectrometry analysis in samples containing uncross-linked PKCε (PKCε) vs samples containing PKCε cross-linked to 14-3-3 (PKCε-14-3-3). (**I**) The percentage of the different types of V3 domain PKCε derived phosphopeptides identified in the uncross-linked and 14-3-3 cross-linked sampled.

The assembly of PKCε with a 14-3-3 dimer has been shown to require the phosphorylation of three sites in the V3 domain of PKCε ([27]; see Figure 1A). We confirmed that assembly was required for the effective completion of cytokinesis in HEK293T cells where PKCε is constitutively engaged [27], through rescue experiments following knockdown of the endogenous PKCε (Figure 1B, Supplementary Figure S1A,B). Unlike ectopic expression of WT PKCε, expression of the 14-3-3 binding incompetent PKCε S346/368A mutant, like the kinase-inactive mutant D532N (the conserved catalytic aspartate), failed to rescue cell division following endogenous PKCε knockdown and the consequent accumulation of binucleate cells.

It has been shown that PKCε control of Aurora B S227 phosphorylation is a key event in exiting from the abscission checkpoint in many transformed cell lines [24] and hence we determined if the assembly of the PKCε/14-3-3 complex was a requirement for Aurora B S227 phosphorylation. It was found that PKCε dependent Aurora B S277 phosphorylation showed the same pattern of dependency as the binucleation phenotype — the WT protein rescued PKCε knockdown, but neither the kinase-dead nor the S346/368A mutant could rescue phosphorylation of Aurora B S227 at the midbody (Figure 1C,D). This connects the assembly of this 14-3-3 complex directly to PKCε action on the midbody-associated Aurora B and successful exit from the abscission checkpoint.

### PKCε S350 is dephosphorylated for 14-3-3 complex assembly

The requirement for PKCε-14-3-3 complex formation in the Aurora B abscission checkpoint, prompted an evaluation of this complex in cells, specifically addressing the nature of the assembled complex. Previous studies had identified a S346/368 bis-phosphorylated V3 domain peptide as a high-affinity binding partner for 14-3-3ζ with the triply phosphorylated species (sites: 346 350 368) displaying a much lower affinity for the 14-3-3ζ dimer [30]. The compromised affinity of the 14-3-3ζ dimer when S350 is phosphorylated was confirmed using an anisotropy approach, both for the short 346 versus 346/350 phosphorylated monovalent interaction peptides, as well as for the longer bivalent interaction peptides retaining 346/368 and 346/350/368 phosphorylated sites (Supplementary Figure S1C,D). This lower affinity might reflect a physiological requirement ensuring greater turnover of the complex or that the priming S350 site, while required to create the docking site for GSK3 and phosphorylation of S346 [27], is in fact dephosphorylated prior to complex assembly. Antisera directed at phosphorylated S350 were not capable of recognising the doubly phosphorylated form of PKCε (346/350) and hence could not resolve this issue (data not shown).

To address the state of phosphorylation of the PKCε-14-3-3 complex from cells, we developed and characterised a cross-linking assay employing a genetically encoded cross-linker (see [29]) directed to the ‘edge’ of the 14-3-3 recognition sequence surrounding S368 [30], specifically residue N371 (see Figure 1E). UV exposure of Abk-modified N371 GFP-PKCε expressed in HEK293Tcells led to the appearance of an immunoreactive protein at 170 kDa, the presumptive size of a GFP-PKCε/14-3-3 complex, that was not observed if either the 346 site or its priming 350 site were mutated (Figure 1F). Probing for S346 phosphorylation revealed both the WT protein and the cross-linked species, but not the S346A nor the S350A species (Figure 1G). This is consistent with the requirement for S346 phosphorylation for complex formation and the dependence of S346 phosphorylation on S350 priming [27]. The cross-linked PKCε species was immunoreactive with a pan-14-3-3 antibody, confirming the identification of a cross-linked 14-3-3 complex (Supplementary Figure S1E). To assess the relevance of this cross-linked complex to the abscission checkpoint, we synchronised GFP-PKCε N371Abk expressing HEK293Tcells with nocodazole and released for 90 min to enable cells to reach late telophase/cytokinesis before UV exposure. Here again, the same cross-linked species is observed (Supplementary Figure S1F).

Exploiting this cross-linking protocol, we compared by mass spectrometry V3 domain derived phosphopeptides from SDS–PAGE gel purified, cross-linked and uncross-linked PKCε. This revealed that for the cross-linked species of PKCε the V3 domain was exclusively phosphorylated on S346 and not S350; by contrast, the uncross-linked species was phosphorylated on both S346 and S350 (Figure 1H,I, Supplementary Figure S1G,H), as well as on S346 + T349 (see Discussion). The complete lack of phosphorylation on S350 in the cross-linked complex demonstrates that this site must be dephosphorylated prior to assembly; it is emphasised that S350 phosphorylation is a prerequisite for S346 phosphorylation (see above Figure 1G and [27]).

### 14-3-3 Complex formation is not required for midbody association and retention

The requirement for PKCε-14-3-3 complex assembly for Aurora B S227 phosphorylation at the midbody and the operation of the abscission checkpoint, suggested that complex assembly might control the recruitment to or the documented activity-dependent retention of PKCε in the midbody region. Previously, it had been shown that inhibition of PKCε catalytic activity led to its accumulation at the midbody during cytokinesis [27], either through cytoplasmic retention or midbody release. To better distinguish these possibilities, we assessed whether the midbody recruitment/retention displayed characteristics distinctive from those evident in a plasma membrane context by determining the rate of signal recovery after photobleaching under conditions of inhibition of NaPP1-sensitive GFP-PKCε M486A. This indicated that the fast component of recovery was considerably slower at the midbody compared with the plasma membrane recruited protein (Supplementary Figure S2A,B). Given the pool of cytoplasmic protein is shared, this indicates that there is an additional property involved in the retention of PKCε at the midbody, i.e. a midbody rather than a cytoplasmic explanation is relevant to this midbody-specific, inhibitor dependent process.

**Figure 2.**
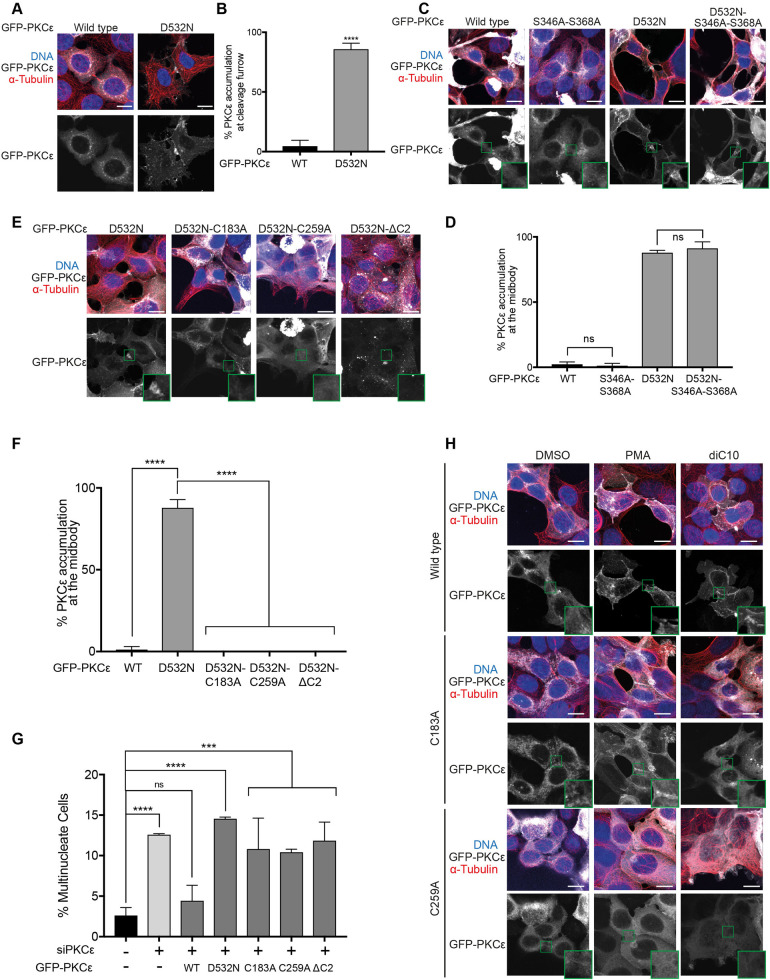
C1 and C2 domains are required for retention and/or recruitment of PKCε to the midbody region. (**A**) Representative immunofluorescence images of HEK293T cells expressing GFP-PKCε and GFP-PKCε-D532N. Cells stained for DNA (blue) and tubulin (red), with PKCε or PKCε-D532N shown in grey. Scale bar = 20 µm. (**B**) Quantitation of dividing cells with GFP-PKCε and GFP-PKCε-D532N accumulation at the midbody. Values plotted are means for *n *= 3 experiments, with error bars denoting SD. Thirty cells with midbodies were observed per condition per experiment. (**C**) Representative immunofluorescence images of dividing HEK293T cells expressing GFP-PKCε, GFP-PKCε-S346A-S368A, GFP-PKCε-D532N or GFP-PKCε-D532N-S346A-S368A. Cells stained for DNA (blue) and tubulin (red), with GFP-PKCε shown in grey; the midbody region highlighted is expanded for each image. Scale bar = 20 µm. (**D**) Quantitation of dividing cells with GFP-PKCε, GFP-PKCε-S346A-S368A, GFP-PKCε-D532N or GFP-PKCε-D532N-S346A-S368A accumulation at the midbody. Values plotted are means for *n *= 3 experiments, with error bars denoting SD. Thirty cells with midbodies were observed per condition per experiment. (**E**) Representative immunofluorescence images of dividing HEK293T cells expressing GFP-PKCε-D532N, GFP-PKCε-D532N-C183A, GFP-PKCε-D532N-C259A or GFP-PKCε-D532N-ΔC2. Cells stained for DNA (blue) and tubulin (red), with GFP-PKCε shown in grey; the midbody region highlighted is expanded for each image. Scale bar = 20 µm. (**F**) Quantitation of dividing cells with GFP-PKCε-D532N, GFP-PKCε-D532N-C183A, GFP-PKCε-D532N-C259A or GFP-PKCε-D532N-ΔC2 accumulation at the midbody. Values plotted are means for *n *= 3 experiments, with error bars denoting SD. Thirty cells with midbodies were observed per condition per experiment. (**G**) Quantitation of multinucleate cells upon treatment with non-targeting siRNA (control) or PKCε siRNA, with expression of siRNA-resistant mouse GFP-PKCε, GFP-PKCε-D532N, GFP-PKCε-C183A, GFP-PKCε-C259A or GFP-PKCε-ΔC2. Values plotted are means for *n *= 3 experiments, with error bars denoting SD. Two-hundred cells were counted per condition per experiment. (**H**) Representative immunofluorescence images of HEK293T cells expressing GFP-PKCε, GFP-PKCε-C183A or GFP-PKCε-C259A and treated with DMSO, PMA or diC10. Cells were treated for 20 min with 1 µM PMA, or 3 min with 10 µM diC10. Cells stained for DNA (blue) and tubulin (red), with GFP-PKCε shown in grey; the midbody region highlighted is expanded for each image. Twenty cells with midbodies were observed per condition across three experiments. Scale bar = 20 µm.

To circumvent any off-target effects of inhibitors in assessing the properties of this activity-dependent localisation and retention, the behaviour of an inactive D532N mutant of PKCε was investigated. The PKCε D532N mutant was found to be constitutively present at the midbody region of dividing cells, while the active GFP-WT fusion does not accumulate there (Figure 2A,B), indicating that the previously documented inhibitor-driven retention of the active protein [27] is a protein autonomous property (note, this behaviour is observed in the absence of knockdown of endogenous PKCε). The D532N mutant thus affords a context in which to test domain mutations and assess their requirements for midbody recruitment and retention without recourse to inhibitors and the potential for confounding non-specific responses. Interestingly, mutations in the V3 domain phosphorylation sites bound by 14-3-3 (S346,368A) alone or when combined with D532N had no impact on the accumulation in the midbody (Figure 2C,D). This indicates that 14-3-3 complex formation is not the driver for PKCε recruitment or retention in this region, despite its assembly being required for Aurora B S227 phosphorylation at the midbody.

### Domain requirements for PKCε recruitment to the midbody

The insensitivity to 14-3-3 complex formation for PKCε recruitment and retention at the midbody region, indicated that other domains are critical for these processes. The C1 domains responsible for DAG binding rely on critical cysteine residues for their function [31]. These cysteines are required for the coordination of Zn^2+^ and their mutation will lead to a loss of domain integrity [32]. Mutating these cysteines in either of the C1A (C183A) or C1B (C259A) domains compromised recruitment/retention of PKCε D532N (Figure 2E,F). Similarly, the deletion of the C2 domain also prevented the recruitment/retention of PKCε D532N (Figure 2E,F). Consistent with this behaviour, the introduction of these mutations into the otherwise WT, active protein, was found not to rescue the knockdown of endogenous PKCε, with multinucleate cells accumulating in each case (Figure 2G, Supplementary Figure S2A,B).

The loss of recruitment with C1 domain mutations suggested that diacylglycerol (DAG) binding might contribute to midbody recruitment. To assess this, cells expressing the C1 domain mutants of wild-type PKCε were treated with the membrane-permeant DAG, DiC10 or phorbol myristate acetate (PMA) an alternative high-affinity C1 domain ligand. In cells undergoing cytokinesis, WT PKCε is recruited to the midbody region on exposure to either of these treatments (Figure 2H, upper panels), indicating that promoting C1 domain-dependent interactions is sufficient to push the steady-state distribution towards recruitment, despite retaining catalytic activity. The enrichment of PKCε at the midbody under these conditions is particularly notable as it occurs in spite of the non-specific nature of lipid or phorbol ester distribution. For the C1A mutation (C183A), PMA still promoted midbody accumulation, consistent with the PKCε C1B high affinity for phorbol esters [33]. Whilst these cysteine mutants may lead to a loss of domain integrity, it is noted that the retention of PMA responsiveness in the C1A only mutated protein provides evidence for the retention of C1B domain integrity reflecting independent folding of these domains. Contrasting with PMA, DiC10 did not recruit the C1A mutant nor the C1B mutant (0% of cells displayed recruitment for these mutations) indicating that DAG-dependent recruitment likely demands the concerted engagement of both of these DAG binding C1 domains, which it is noted display similar affinities for DAG [34]. The C1B domain mutant (C259A) was also not recruited by PMA, consistent with the lower affinity for phorbol esters reported for the residual PKCε C1A domain (Figure 2H; [33]). This pattern of C1 domain behaviour reflects known properties and is indicative of a DAG-dependent element in the recruitment to the midbody region. The finding that DAG alone was sufficient to enrich PKCε at the midbody region signifies that exogenous DAG, which distributes widely in the membranes of cells, is not necessarily enriched at the midbody to trigger PKCε enrichment and that the working model of membrane recruitment and selective (activity sensitive) retention at the midbody is a consistent view of events compatible with the FRAP data above. The PKCεΔC2 mutant was assessed also and interestingly it was found to be recruited by either PMA (Supplementary Figure S2E) or DiC10 (data not shown), indicating that C2 domain deletion does not compromise the C1 domain-dependent recruitment; this suggests that the C1 domain itself may contribute specifically to protein–protein interactions and the localised retention (reviewed [35]).

**Figure 3.**
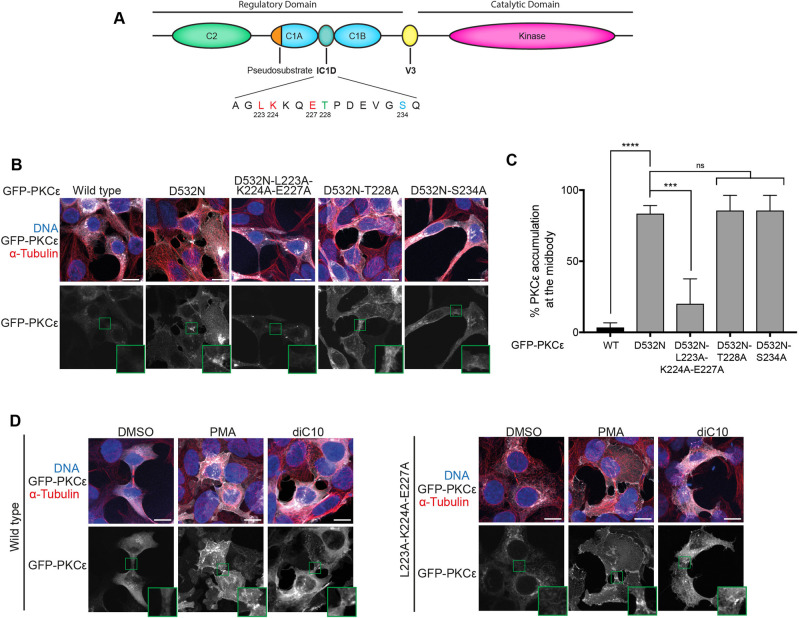
The IC1D domain is necessary for retention but not recruitment to the midbody region. (**A**) Schematic representing the organisation of PKCε, with the residues of the Inter-C1 Domain (IC1D) noted with the three residues that make up the actin binding motif in red, and the two phosphorylation sites in blue and green. (**B**) Representative immunofluorescence images of dividing HEK293T cells expressing GFP-PKCε, GFP-PKCε-D532N, GFP-PKCε-D532N-L223A-K224A-E227A, GFP-PKCε-D532N-T228A or GFP-PKCε-D532N-S234A. Cells stained for DNA (blue) and tubulin (red), with GFP-PKCε shown in grey; the midbody region highlighted is expanded for each image. Scale bar = 20 µm. (**C**) Quantitation of dividing cells with GFP-PKCε, GFP-PKCε-D532N, GFP-PKCε-D532N-L223A-K224A-E227A, GFP-PKCε-D532N-T228A or GFP-PKCε-D532N-S234A accumulation at the midbody. Values plotted are means for *n *= 3 experiments, with error bars denoting SD. Thirty cells with midbodies were observed per condition per experiment. One-way analysis of variance: **** = *P *≤ 0.0001; *** = *P *≤ 0.001; ns (not significant) = *P *> 0.05. (**D**) Representative immunofluorescence images of HEK293T cells expressing GFP-PKCε, GFP-PKCε-D532N or GFP-PKCε-L223A-K224A-E227A and treated with DMSO, PMA or diC10 as indicated. Cells were treated for 20 min with 1 µM PMA, or 3 min with 10 µM diC10. Cells stained for DNA (blue) and tubulin (red), with GFP-PKCε shown in grey; the midbody region highlighted is expanded for each image. Twenty cells with midbodies were observed per condition across three experiments. Scale bar = 20 µm.

Deletion of the inter-C1 domain (IC1D) has been reported to prevent midbody localisation of PKCε [36]. Given the apparent requirement for C1A/B domains in this recruitment, it was possible that altered conformation in this region might trigger this loss of recruitment indirectly, by interfering with the C1A/B domain function. The IC1D has been reported to be an actin-binding region for PKCε [37], centred upon the residues L223/K224/E227 (Figure 3A). To compromise the IC1D without deletion, an LKE > 3A triple mutant was tested for recruitment in the context of the D532N mutation. The LKE > 3A mutant was not constitutively retained at the midbody (Figure 3B,C). However, on exposure to PMA or DiC10 the LKE > 3A mutant was recruited to the midbody (Figure 3D). This indicates that functional C1A/B domains are retained by this mutant and that the protein is competent in respect of recruitment, but by inference compromised in part with respect to retention. The IC1D also retains two phosphorylation sites, T228 and S234, but neither an S234A,D532N nor a T228A,D532N mutant impacted recruitment/retention (Figure 3B,C) and hence these sites do not appear to account for the activity-dependent behaviour. It is also noted that in log phase cultures (i.e. encompassing all cell cycle phases) AbK sidechain-encoded cross-linking studies (as for 14.3.3 above) have failed to identify actin as a binding partner for this domain and an unbiased mass spectrometry approach has likewise failed to identify a partner cross-linked through this domain that impacts midbody retention (LW and PJP, unpublished).

### The IC1D is required for Aurora B S227 phosphorylation and control of the abscission checkpoint

**Figure 4.**
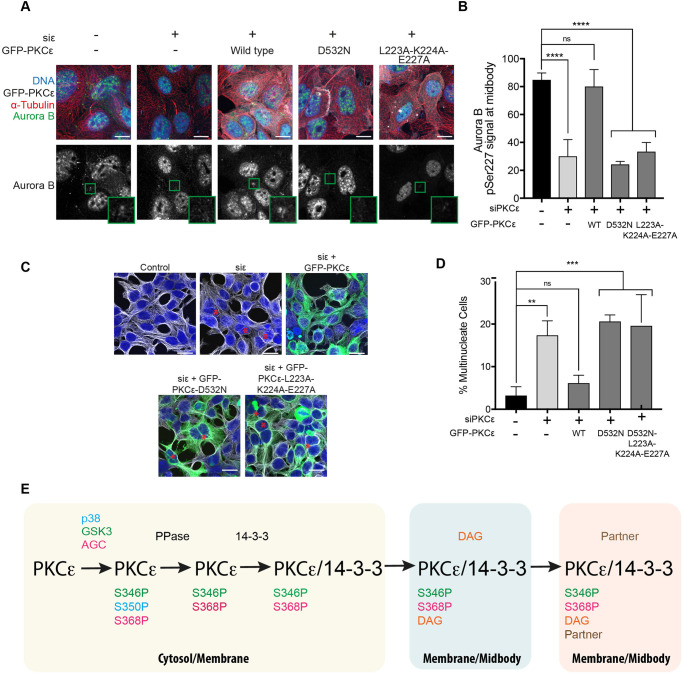
Retention at the midbody region is required for Aurora B S227 phosphorylation. (**A**) Representative immunofluorescence images of DLD1 FRT-TREx cells treated with non-targeting siRNA (control) or PKCε siRNA, with expression of either siRNA-resistant mouse GFP-PKCε, GFP-PKCε-D532N or GFP-PKCε-L223-K224-E227A induced with doxycycline. Cells stained for DNA (blue), tubulin (red) and Aurora B pSer227 (green), with GFP-PKCε in white if expressed. The Aurora B S227 phosphorylation is highlighted in the lower greyscale panels with the midbody region indicated expanded for each image. Scale bar = 20 µm. (**B**) Quantitation of cells with Aurora B pSer227 signal at the midbody. DLD1 FRT-TREx cells with inducible expression of GFP-PKCε, GFP-PKCε-D532N or GFP-PKCε-L223-K224-E227A were treated with either non-targeting siRNA (control), PKCε siRNA or PKCε siRNA and doxycycline to induce expression of siRNA-resistant mouse GFP-PKCε constructs, and the Aurora B pSer227 signal at the midbody quantified. Values plotted are means for *n *= 3 experiments, with error bars denoting SD. Twenty cells with midbodies were counted per condition per experiment. One-way analysis of variance: **** = *P *≤ 0.0001; ns (not significant) = *P *> 0.05. (**C**) Representative immunofluorescence images of HEK293T cells treated with non-targeting siRNA (control) or PKCε siRNA, with expression of siRNA-resistant mouse GFP-PKCε, GFP-PKCε-D532N or GFP-PKCε-L223-K224-E227A. Cells stained for DNA (blue) and tubulin (grey), with GFP-PKCε in green. Scale bar = 10 µm; red asterisks indicate multinucleate cells. (**D**) Quantitation of multinucleate cells upon treatment with non-targeting siRNA (control) or PKCε siRNA, with expression of siRNA-resistant mouse GFP-PKCε, GFP-PKCε-D532N or GFP-PKCε-L223-K224-E227A. Values plotted are means for *n *= 3 experiments, with error bars denoting SD. Two-hundred cells were counted per condition per experiment. One-way analysis of variance: *** = *P *≤ 0.001; ** = *P *≤ 0.01; ns (not significant) = *P *> 0.05. (**E**) Working model of PKCε turnover at midbody and mediation of exit from the abscission checkpoint. The different states of PKCε are indicated alongside the involved upstream regulators. Predicted compartments are indicated by coloured boxes. The endpoint of the partner bound, membrane associated, 14-3-3 complex is the form of PKCε required for Aurora B S227 phosphorylation at the midbody and exit from the abscission checkpoint.

The DAG-competent recruitment of the PKCεLKE > 3A mutant and the lack of retention on expression as an inactive form (D532N), provides a means with which to address the requirement for the activity regulated retention of PKCε in the context of the abscission checkpoint. Expression of the LKE > 3A mutant retained the ability to form a 14-3-3 complex as this mutant cross-linked exactly as the WT protein (Supplementary Figure S3A). Significantly, the expression of this mutant failed to support Aurora B S227 phosphorylation at the midbody (Figure 4A,B). This correlated with an inability to rescue knockdown of endogenous PKCε and suppress division failure (Figure 4C,D). Together, these observations support a model where PKCε midbody recruitment, retention and 14-3-3 complex assembly act in concert to control Aurora B and the abscission checkpoint (Figure 4E).

## Discussion

While there is strong evidence for PKCε action in determining exit from the abscission checkpoint [24,27], there has been limited insight into its recruitment and retention in relation to its action at the midbody region. The investigation here indicates that there are at least three independent elements to this, each of which appears to be necessary to drive the PKCε dependent Aurora B S227 phosphorylation at the midbody required for checkpoint exit, protecting from division failure and consequent binucleation. This is unexpected given the prior findings that the PKCε-14-3-3 complex was essential for checkpoint exit and that the properties associated with this complex indicated that it was activated [27], i.e. there is no a priori requirement for more typical lipid-activating inputs. What might the multiple inputs reflect in terms of the mechanism? As a working model to inform further analysis, it is proposed that the 14-3-3 complex is formed through the combined action of upstream kinases and phosphatases (see Figure 4E), independently of any specific recruitment to the midbody region. This is informed by the distinct contexts in which this complex can form independent of cytokinesis [38], the observed pre-anaphase S346 phosphorylation [27] and the competence of the midbody non-retained IC1D mutant to be cross-linked to 14-3-3 (see Figure 4). The complex is then recruited to the midbody region through C1 domain interactions with DAG, under conditions where the level of DAG is not sufficient to sustain the PKCε-14-3-3 complex in this compartment; artificially elevating the levels of DAG trigger a more sustained recruitment directly. Subsequent association of the membrane recruited complex with a midbody-localised partner(s) through C1/IC1D/C2 interactions ‘holds’ the complex at the midbody, but can be dissociated by PKCε activity giving rise to the inhibition dependent accumulation seen at endogenous DAG levels. As a final rationalisation of the data and specifically the requirement for 14-3-3 complex formation, it is surmised that this lipid-independent active midbody complex may need to dissociate from the membrane to act locally upon the midbody bound Aurora B.

The normal development and maturation of the PKCε knockout mouse indicates that there is no absolute requirement for this kinase in the process of cell division *per se*. For this reason, the specific engagement of PKCε in the control of Aurora B is anticipated to be linked to exceptional, stress-associated events and not to routine cell division. In relation to this, we note that the engagement of PKCε in these controls is only evident in cells where there is a defect (somatic or induced) in the Topo2a-dependent G2 arrest [21,24]. The implication is that progression through G2 despite chromatin-stressed circumstances exacerbated/reproduced by chromatin-associated Topo2a, triggers dependence on PKCε. In respect of AuroraB and the abscission checkpoint, this conditional engagement of PKCε indicates that either the S227 site on AuroraB is targeted by another kinase under normal circumstances and that there is the general engagement of the abscission checkpoint, or that the engagement of the abscission checkpoint is rarely implemented under normal circumstances. The notion that the AuroraB abscission checkpoint acts to determine the timing of abscission as part of the normal programme of events (reviewed in [39]), is in part based upon studies carried out in transformed cells where the Topo2-dependent G2 arrest is frequently lost and there is a constitutive engagement of the abscission checkpoint (for example in Hela cells; [5,6,19,25,40]). It remains to be seen where and when this is the case for ‘normal’ cells where PKCε has no impact on these cell cycle processes.

In cells with a defective Topo2-dependent G2 arrest response, there is evidence for a PKCε-Aurora B module to impact the metaphase–anaphase transition [22]. Here, the focus on 14-3-3 complex formation and midbody recruitment addresses specifically events later in the cycle; V3 domain phosphorylation and hence 14-3-3 complex formation is not involved in the actions of PKCε in M-Phase [22]. The exit from the Aurora B abscission checkpoint is often described as requiring inactivation of Aurora B. However, in the original description of this pathway's operation in mammalian cells, Aurora B activity is reported to be required to trigger the checkpoint and to maintain its activity to stabilise the intercellular canal that forms. Inhibition following engagement of the checkpoint leads to furrow regression, division failure and polyploidisation [6]; inhibition at this late stage does not facilitate completion of cytokinesis. Endogenously, the timing of the exit from this checkpoint appears to be regulated in part by PKCε through its control of Aurora B. This is not by simply inactivating, rather, S227 phosphorylation switches Aurora B substrate specificity, consequently promoting Aurora B phosphorylation of Borealin S165 a critical downstream event in this exit [24] and required to regulate CHMP4C conformation and ESCRTIII-complex formation [5,40] to facilitate the final scission event. How the dynamic behaviour of PKCε at the midbody acts to drive Aurora B S227 phosphorylation at the right time remains to be determined, as indeed does the sensing mechanism that defines the right time. For Aurora B S227 phosphorylation, the timing might reflect a gain of opportunity in respect of PKCε-14-3-3/Aurora B proximity impacted by a shift in the steady-state occupancy of the midbody compartment or perhaps the influence of protein phosphatases acting on the S227 site; both PP1 and PP2A display multiple, location-specific roles in determining cell cycle progression (recently reviewed [41]) and more specifically, PP1 recruitment has been shown to contribute to exit timing through the dephosphorylation of CHMP4C [25].

The working model above, aligns with the idea of receptors for activated C-kinases (RACKS) first elaborated by Mochly-Rosen et al. [42]. A proposed function of RACKS is to hold active conformations of PKC family proteins in specific compartments (see review [43]). There are no well-understood examples of the action of such RACKS, so the actions here might provide a well-defined context in which such an interaction plays a crucial role. The identity of the midbody associating protein will no doubt bring clarity to this possibility. It is of interest that PKC partner proteins such as AKAPs (reviewed [44]) display phosphorylation-dependent associations as exemplified by the association of AKAP149 with PP1 [45]. AKAP12 (also referred to as Gravin/SSeCKS) has been reported to localise to the contractile ring and control cytokinesis [46] and is a known interactor of PKC family members (see [47]). However preliminary evidence suggests that knockdown of this scaffold protein does not impact the activity-dependent retention of PKCε at the midbody (LW, JDS and PJP unpublished). Apparently, there is another PKCε partner responsible for retention of the kinase in the midbody region.

The finding that PKCε has to be dephosphorylated prior to assembly of the 14-3-3 complex is interesting. The phosphorylation of the S346 site by GSK3 requires prior phosphorylation at the S350 site [27], consistent with the known recognition specificity of this kinase [48]. The weakening of the S346 recognition by 14-3-3 on retention of the S350 site evidently is sufficient to preclude complex formation in cells, hence the exclusive S346 phosphorylation at this site in the cross-linked complex. How the dephosphorylation of PKCε S350 is executed remains to be determined. Initial studies have indicated that recombinant cdc14 from yeast can dephosphorylate this site in peptide assays; however, there is no evidence that cdc14 is responsible for this dephosphorylation in cells (LW and PJP, unpublished). It is of interest to note that there are few other documented examples of GSK3-dependent phosphorylations promoting 14-3-3 complex assembly where sites have been mapped definitively. In the case of RASSF1, GSK3 has been shown to be required for basal state complex assembly through phosphorylation on 175/178/179 [49]. Based upon the behaviour documented here, it seems unlikely that 14-3-3 will interact with RASSF1 directly through these sites if they are fully phosphorylated. One additional observation here for PKCε is the identification of the doubly phosphorylated sequence comprising the sites S346 and T349. This was found exclusively in the non-cross-linked protein, indicating that the presence of phospho-T349 is not compatible with 14-3-3 recognition of the phosphorylated 346 residues. This is entirely consistent with the structure determined for the V3 domain-14-3-3 complex [30]. While S350 is required as a docking site for GSK3 to enable S346 phosphorylation, T349 phosphorylation likely occurs after S350 dephosphorylation given the occupation of S346. It will be of interest to determine the extent to which the phosphorylation of T349 is exploited to actively block complex formation and determine the timing of exit from the checkpoint.

There is a long history of association between PKCε and transformation, from the very earliest studies reported by Weinstein's laboratory on oncogenic properties in culture [50], to the recent demonstration of a PKCε requirement in KRas-induced transformation *in vivo* [51]. These studies broadly address the promotion of properties typically associated with cancer and offer a druggable opportunity for intervention. In contrast, the behaviour investigated here relates to the engagement of PKCε in a failsafe programme and unlike the oncogenic driver behaviour, this is in effect protective of properties of a subset of oncogenic states (determined experimentally by the activity/inactivity of the Topo2-dependent G2 arrest [21]). Inhibition of PKCε in this context is not a direct attack on the drivers of disease but instead targets a tumour-specific vulnerability, resulting in a synthetic lethal outcome, as a function of the G2 arrest competence (discussed [52]). The apparent normality of the PKCε knockout mouse [53,54], suggests that PKCε intervention in the right context would afford an excellent therapeutic index.

In conclusion, it is demonstrated that the PKCε dependent phosphorylation of Aurora B and exit from the abscission checkpoint is reliant on a series of functions impacting PKCε and its subcellular localisation. The apparent prelude to this is the combined action of three upstream kinases and a phosphatase that enable complex formation with 14-3-3. This is followed by DAG-dependent recruitment and associated retention in the midbody region in a manner that is dependent upon the intrinsic activity of the kinase. If all of these are in place, then Aurora B becomes phosphorylated on S227 and this supports exit from the abscission checkpoint and completion of cell division.

## Data Availability

The mass spectrometry proteomics data have been deposited to the ProteomeXchange Consortium via the PRIDE [55] partner repository with the dataset identifier PXD025959 and 10.6019/PXD025959.

## Open Access

Open access for this article was enabled by the participation of The Francis Crick Institute in an all-inclusive *Read & Publish* pilot with Portland Press and the Biochemical Society under a transformative agreement with JISC.

## Abbreviations

CPC: chromosome passenger complex
FRAP: fluorescence recovery after photobleaching
PD: proteome discover
RACKS: receptors for activated c-kinases
