# Supplementary material for: Co-ordinated control of the Aurora B abscission checkpoint by PKCε complex assembly, midbody recruitment and retention
Source: Biochem J. 2021 Jun 18;478(12):2247–63. doi: 10.1042/BCJ20210283 (PMC8238520; doi:10.1042/BCJ20210283)
Supplement: Supplementary Figures S1-S3 [file BCJ-478-2247-s1.pdf]

Supplementary Figure S1

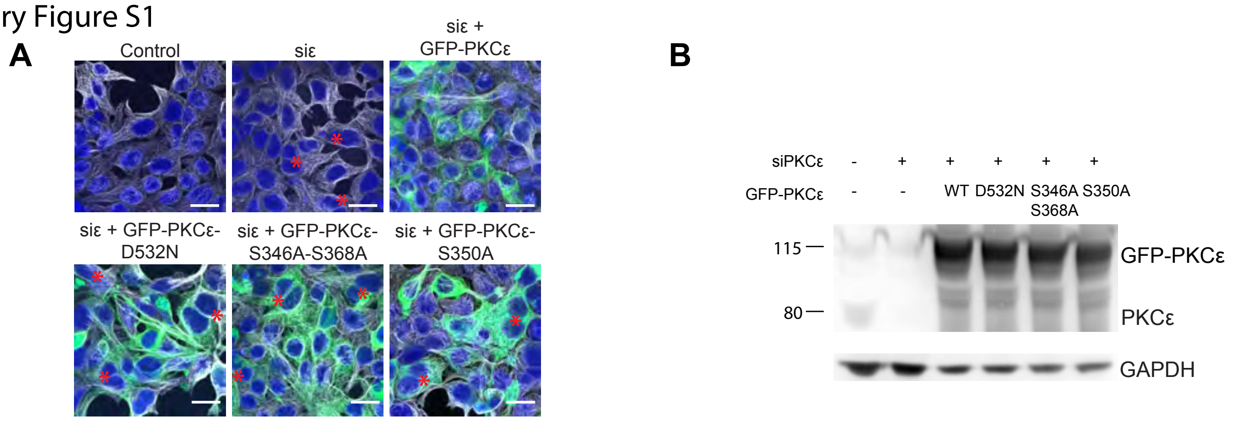

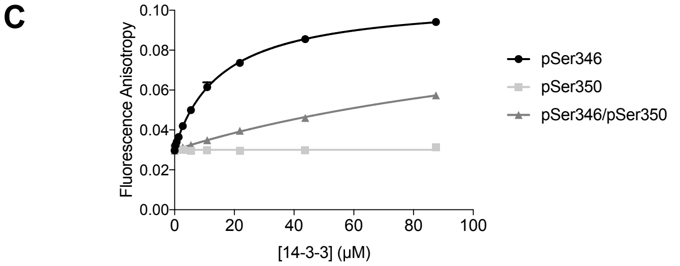

Peptide	K_d Value (μM)	Std. Error
pSer346	31.5	1.5
pSer350	n/a	n/a
pSer346/pSer350	335	43

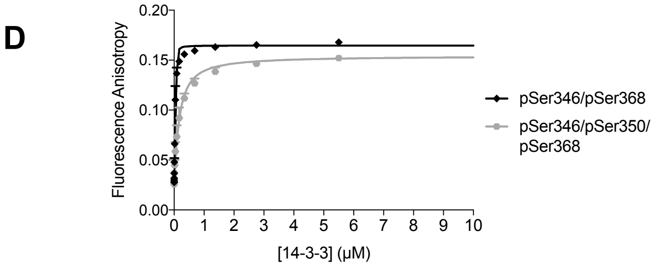

Peptide	K_d Value (μM)	Std. Error
pSer346/pSer368	0.030	0.003
pSer346/pSer350/pSer368	0.147	0.013

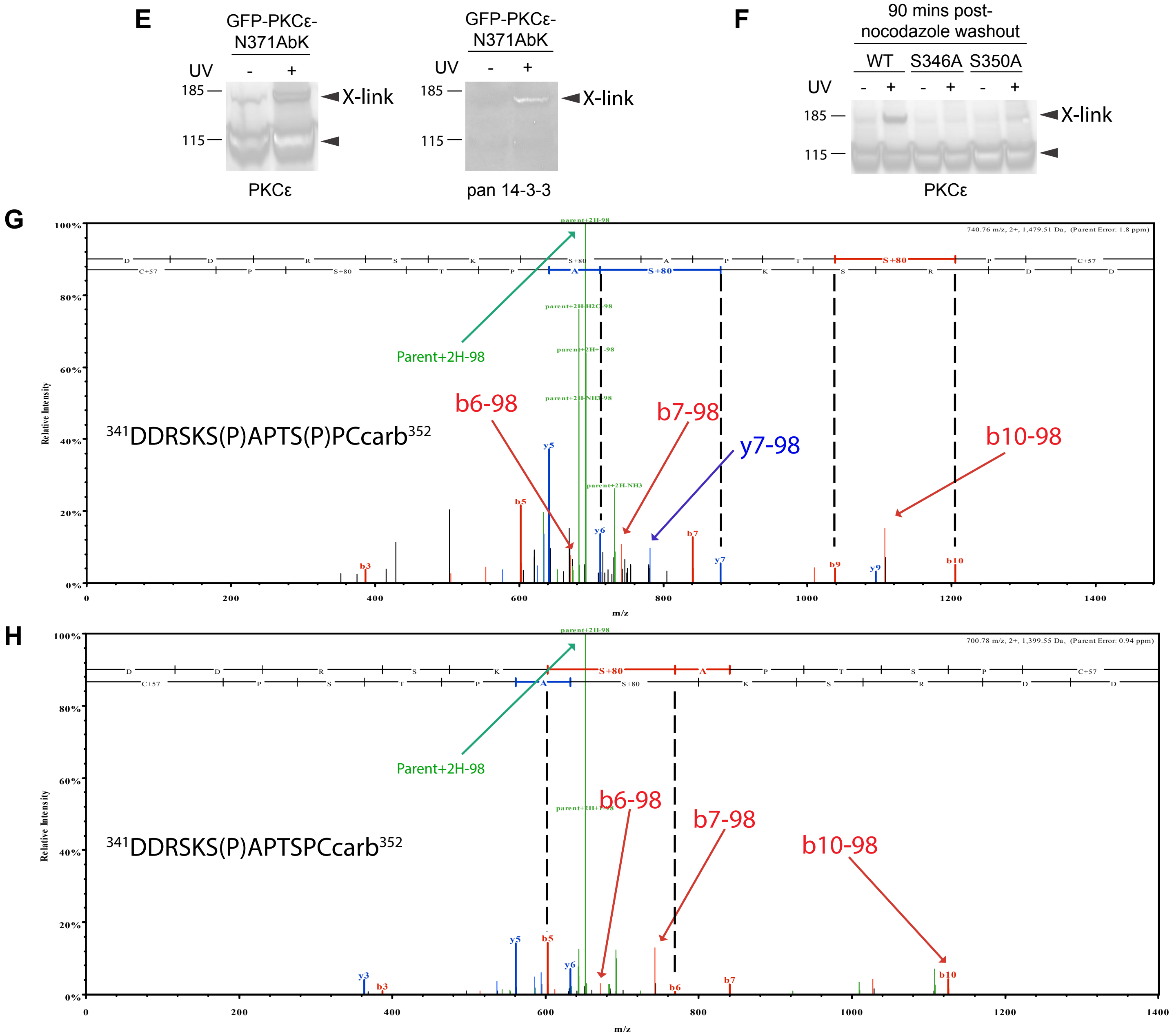

Supplementary Figure 1. Requirements for PKCε-14.3.3 complex formation (A) Representative immunofluorescence images of HEK293T cells treated with non-targeting siRNA (control) or PKCε siRNA, with expression of siRNA-resistant mouse GFP-PKCε, GFP-PKCε-D532N, GFP-PKCε-S346A-S368A or GFP-PKCε-S350A. Cells stained for DNA (blue) and tubulin (grey), with GFP-PKCε in green. Scale bar = 10 µm; red * = multinucleate cell. (B) Western blot probed with PKCε and GAPDH antibodies showing lysates of HEK293T cells treated with non-targeting siRNA (control) or PKCε siRNA, with expression of siRNA-resistant mouse GFP-PKCε, GFP-PKCε-D532N, GFP-PKCε-S346A-S368A or GFP-PKCε-S350A. Western blot image is representative of two independent experiments. (C) Fluorescence polarisation data for the binding of recombinant 14-3-3 to pSer346, pSer350 and pSer346/pSer350 PKCε phosphopeptides. Values plotted are means for n=3 experiments. (D) Fluorescence polarisation data for the binding of recombinant 14-3-3 to pSer346/pSer368 and pSer346/pSer350/pSer368 PKCε phosphopeptides. Concentrations and Kd values refer to the dimer concentration of 14-3-3. Values plotted are means for n=3 experiments. (E) Western blots probed with PKCε antibody and pan 14-3-3 antibody, showing that the crosslinked band obtained upon expression of GFP-PKCε-N371AbK and 365nm UV exposure for 10 minutes is crosslinking of PKCε to 14-3-3. The upper and lower arrowheads indicate cross-linked (X-link) PKCε and PKCε respectively. Western blot image is a representative example of three independent experiments. (F) Western blot probed with PKCε antibody and phosphoSer346 antibody showing HEK293T cells 90 minutes after release from nocodazole synchronisation expressing GFP-PKCε-N371AbK, GFP-PKCε-N371AbK-S346A or GFP-PKCε-N371AbK-S350A and crosslinking occurring when exposed to 365nm UV for 10 minutes. The upper and lower arrowheads indicate cross-linked (X-link) PKCε and PKCε respectively. Western blot image is a representative example of three independent experiments. (G) Mass spectrometry spectra of pSer346 and pSer350 found in the sample containing uncrosslinked PKCε. (H) Mass spectrometry spectra of pSer346 alone found in the sample containing PKCε crosslinked to 14-3-3.

Supplementary Figure S2

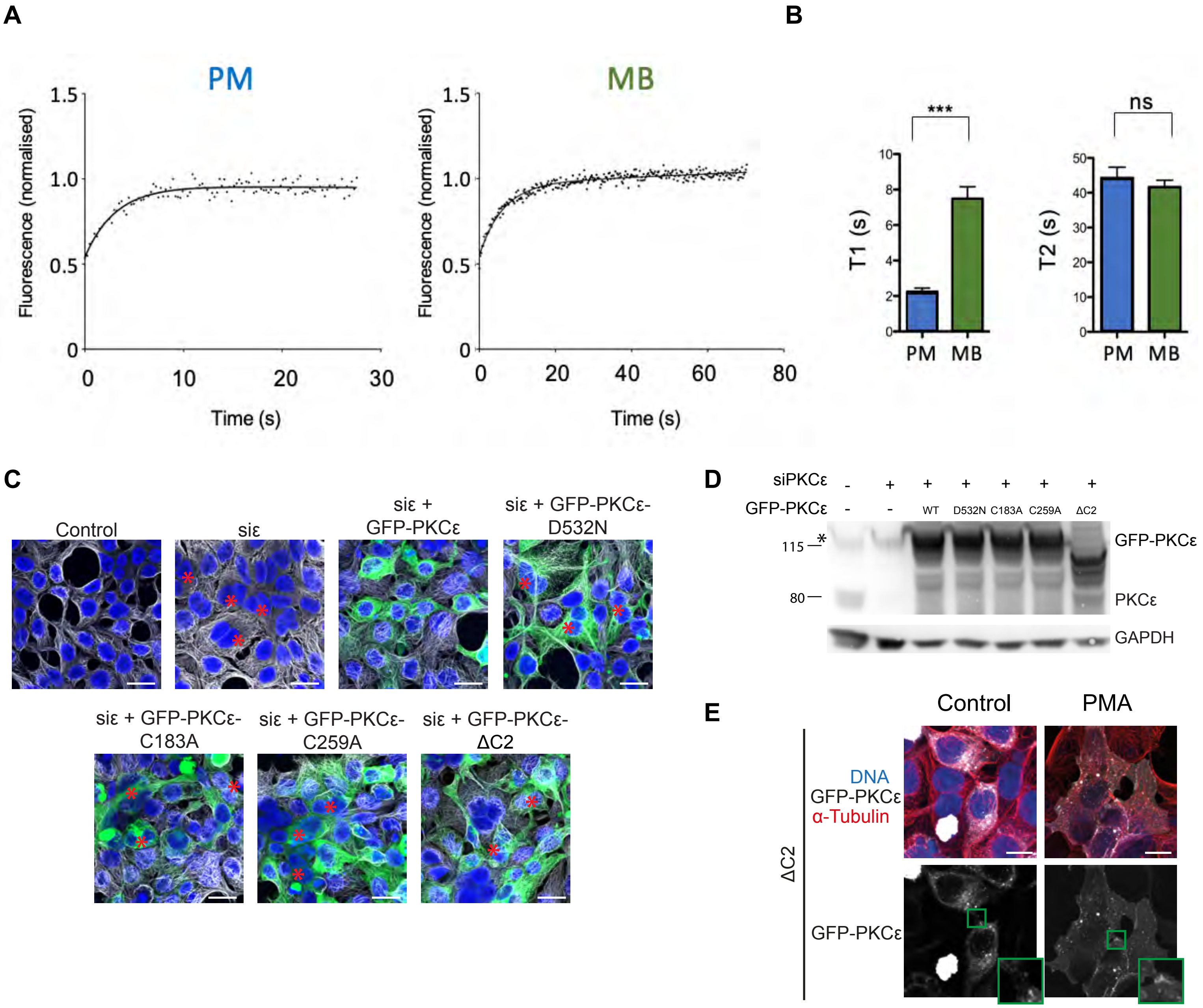

Supplementary Figure 2. Midbody recruitment and retention, and the expression of C1 and C2 domain mutations.

(A) A normalised recovery series fitted to a double exponential model: $I = I_0 - I_1e^{-t/T1} - I_2e^{-t/T2}$ (see Methods), for GFP-PKCε M486A expressing HEK293T cells pretreated with NaPP1. Cells were bleached at the plasma membrane (PM) or midbody (MB) and fluorescence recovery was monitored. (B) The means ± SD of T_1 and T_2 values are shown for plasma membrane (PM) and midbody (MB) recruited GFP-PKCε M486A in the presence of NaPP1. Values were fitted as in (A); data from 20 measurements are presented. There is a significant difference between the T_1 values for membrane and midbody recovery rates ($P<0.001$); no difference is observed for the T_2 component. (C) Representative immunofluorescence images of HEK293T cells treated with non-targeting siRNA (control) or PKCε siRNA, with expression of siRNA-resistant mouse GFP-PKCε, GFP-PKCε-D532N, GFP-PKCε-C183A, GFP-PKCε-C259A or GFP-PKCε-ΔC2. Cells stained for DNA (blue) and tubulin (grey), with GFP-PKCε in green. Scale bar = 10 µm; red asterisks indicate multinucleate cells. (D) Western blot probed with PKCε and GAPDH antibodies showing lysates of HEK293T cells treated with non-targeting siRNA (control) or PKCε siRNA, with expression of siRNA-resistant mouse GFP-PKCε (WT), GFP-PKCε-D532N, GFP-PKCε-C183A, GFP-PKCε-C259A or GFP-PKCε-ΔC2. Western blot image is representative of two independent experiments. The asterisk denotes an immunoreactive non-specific band. (E) Representative images of GFP-PKCε-ΔC2 expressing HEK293T cells treated with DMSO (control) or PMA for 20 minutes. Cells were stained for DNA (blue) and tubulin (red), with GFP-PKCε shown in grey; the midbody region highlighted is expanded for each image. 20 cells were observed per condition across 3 experiments. Scale bar = 20 µm.

Supplementary Figure S3

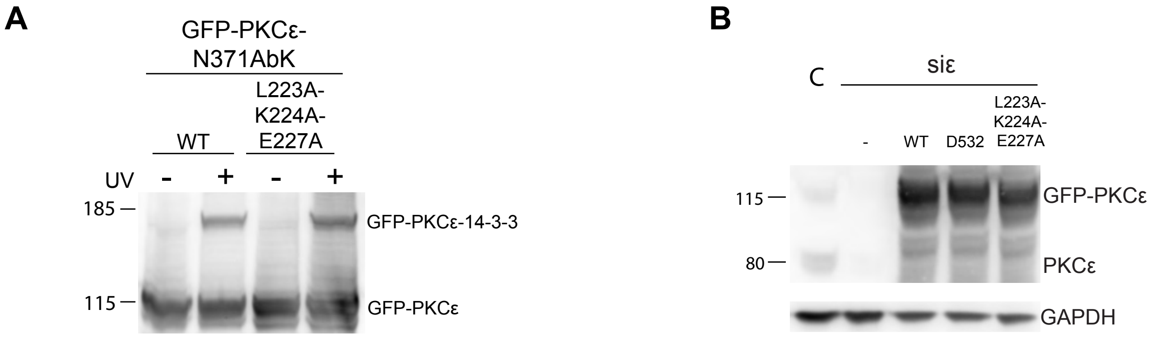

Supplementary Figure 3

(A) Western blot probed with PKCε antibody showing crosslinking of PKCε to 14-3-3 is unchanged upon incorporation of the mutations L223A, K224A and E227A into GFP-PKCε-N371AbK and upon exposure to 365nm UV for 10 minutes. Western blot image is a representative example of two independent experiments. (B) Western blot probed with PKCε and GAPDH antibodies showing lysates of HEK293T cells treated with non-targeting siRNA (control) or PKCε siRNA, with expression of siRNA-resistant mouse GFP-PKCε, GFP-PKCε-D532N or GFP-PKCε-L223-K224-E227A. Western blot image is a representative example of two independent experiments.
